# Positioning Accuracy in Otosurgery Measured with Optical Tracking

**DOI:** 10.1371/journal.pone.0152623

**Published:** 2016-03-30

**Authors:** Attila Óvári, Dóra Neményi, Tino Just, Tobias Schuldt, Anne Buhr, Robert Mlynski, András Csókay, Hans-Wilhelm Pau, István Valálik

**Affiliations:** 1 Department of Oto-Rhino-Laryngology, Head & Neck Surgery, “Otto Koerner”, University Medical Center, Rostock, Germany; 2 Department of Neurology, University Medical Center, Rostock, Germany; 3 Department of Oto-Rhino-Laryngology, KMG Klinikum Güstrow GmbH, Güstrow, Germany; 4 Department of Neurosurgery, Military Hospital, Budapest, Hungary; 5 Department of Neurosurgery, St. John’s Hospital, Budapest, Hungary; Semmelweis University, HUNGARY

## Abstract

**Objectives:**

To assess positioning accuracy in otosurgery and to test the impact of the two-handed instrument holding technique and the instrument support technique on surgical precision. To test an otologic training model with optical tracking.

**Study Design:**

In total, 14 ENT surgeons in the same department with different levels of surgical experience performed static and dynamic tasks with otologic microinstruments under simulated otosurgical conditions.

**Methods:**

Tip motion of the microinstrument was registered in three dimensions by optical tracking during 10 different tasks simulating surgical steps such as prosthesis crimping and dissection of the middle ear using formalin-fixed temporal bone. Instrument marker trajectories were compared within groups of experienced and less experienced surgeons performing uncompensated or compensated exercises.

**Results:**

Experienced surgeons have significantly better positioning accuracy than novice ear surgeons in terms of mean displacement values of marker trajectories. The instrument support and the two-handed instrument holding techniques significantly reduce surgeons’ tremor. The laboratory set-up presented in this study provides precise feedback for otosurgeons about their surgical skills and proved to be a useful device for otosurgical training.

**Conclusions:**

Simple tremor compensation techniques may offer trainees the potential to improve their positioning accuracy to the level of more experienced surgeons. Training in an experimental otologic environment with optical tracking may aid acquisition of technical skills in middle ear surgery and potentially shorten the learning curve. Thus, simulated exercises of surgical steps should be integrated into the training of otosurgeons.

## Introduction

Manipulation during middle ear surgery requires high levels of technical skill and virtually tremor-free handwork. Despite the clinical significance of microsurgical precision in otology, there are little data dealing with the positioning accuracy of otologists [1–3]. Meanwhile, the number of publications about accuracy enhancement devices such as micromanipulators and surgical robots in otology is expanding [2–14].Since most otologists favor a conventional microsurgical training without the application of a tremor-reducing device and work free-handed, it seems to be useful to analyze their performance in improving surgical precision. Therefore, using a simulated otologic environment, we tried to quantify the hand movement accuracy of 14 ENT surgeons in the same department with varying levels of surgical experience in middle ear surgery.

## Materials and Methods

### Ethic statement

This research was conducted according to the principles expressed in the Declaration of Helsinki. Every test person gave written informed consent to participate in this study and this consent was documented in study protocols. Test persons were clinicians with complete medical studies and staff members in the same ENT department. Every test person decided freely to participate in this study and they were generally motivated to test themselves and receive feedback about their surgical skills. Participation, just as any eventual refusal to participate, had no influence in any way on their career development. Furthermore, the performance achieved was not used to classify, rank, promote or disadvantage test persons. Participants performed basic tasks with instruments for otosurgery to simulate routine surgical steps in a laboratory environment. No patients were involved. Test persons’ data were anonymized by a medical technical assistant and final data analysis was performed blinded by two investigators in the study (AC, IV) not knowing the test persons. Five of the test persons (AO, TJ, TS, AB and HP) contributed to the manuscript and matched authorship criteria.

The temporal bone specimen used in this study originated from a willed whole body donation program at the Anatomical Institute of Rostock University. According to German laws, a person wishing to donate his/her whole body for medical research or teaching students after his/her death should register voluntarily with the program during his/her life. Written consent of the donor in this study has ensued in the Anatomical Institute and once registered, the privacy of the donor was protected. None of the participants in this study had any identifying information such as the name, gender or age of the donor, nor had they access to the database of the willed body donation program.

For the reasons mentioned above, approval of the ethics committee was not necessary.

### Temporal bone dissection

Measurements were performed using a specially dissected formalin-fixed human temporal bone, obtained from a body donor via the Anatomical Institute of Rostock University. Bony overhangs on the temporal bone were sawed off and polished to achieve one block with dimensions45×35×20 mm containing the tympanic cavity. Only 5 mm of the medial portion of the bony outer ear canal wall was spared. Middle ear ossicles were also preserved. A surgical situation of a stapesplasty was simulated by exposing the oval window niche with the stapes and long process of the incus.

### Instruments and tasks

**Fig 1:**
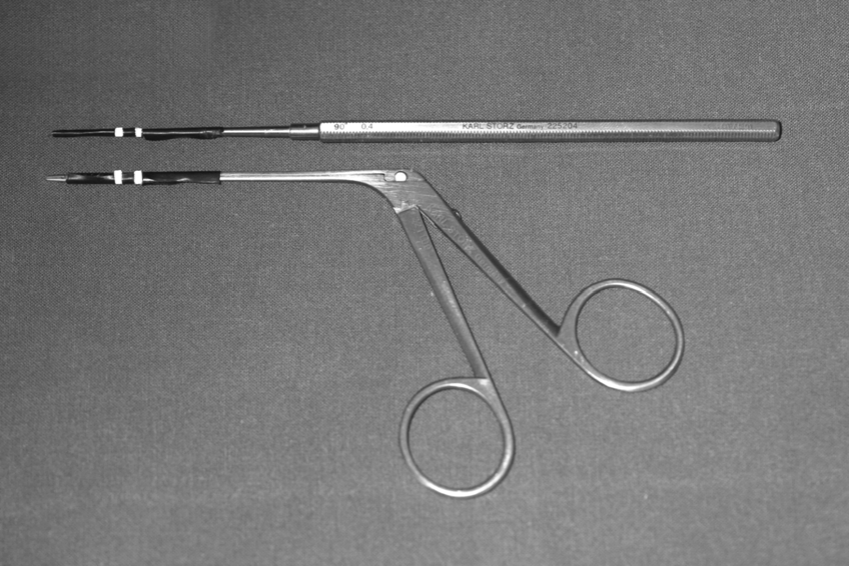
Otologic microinstruments. Otologic microinstruments used in this study (pick and forceps) with markers for optical tracking.

**Fig 2:**
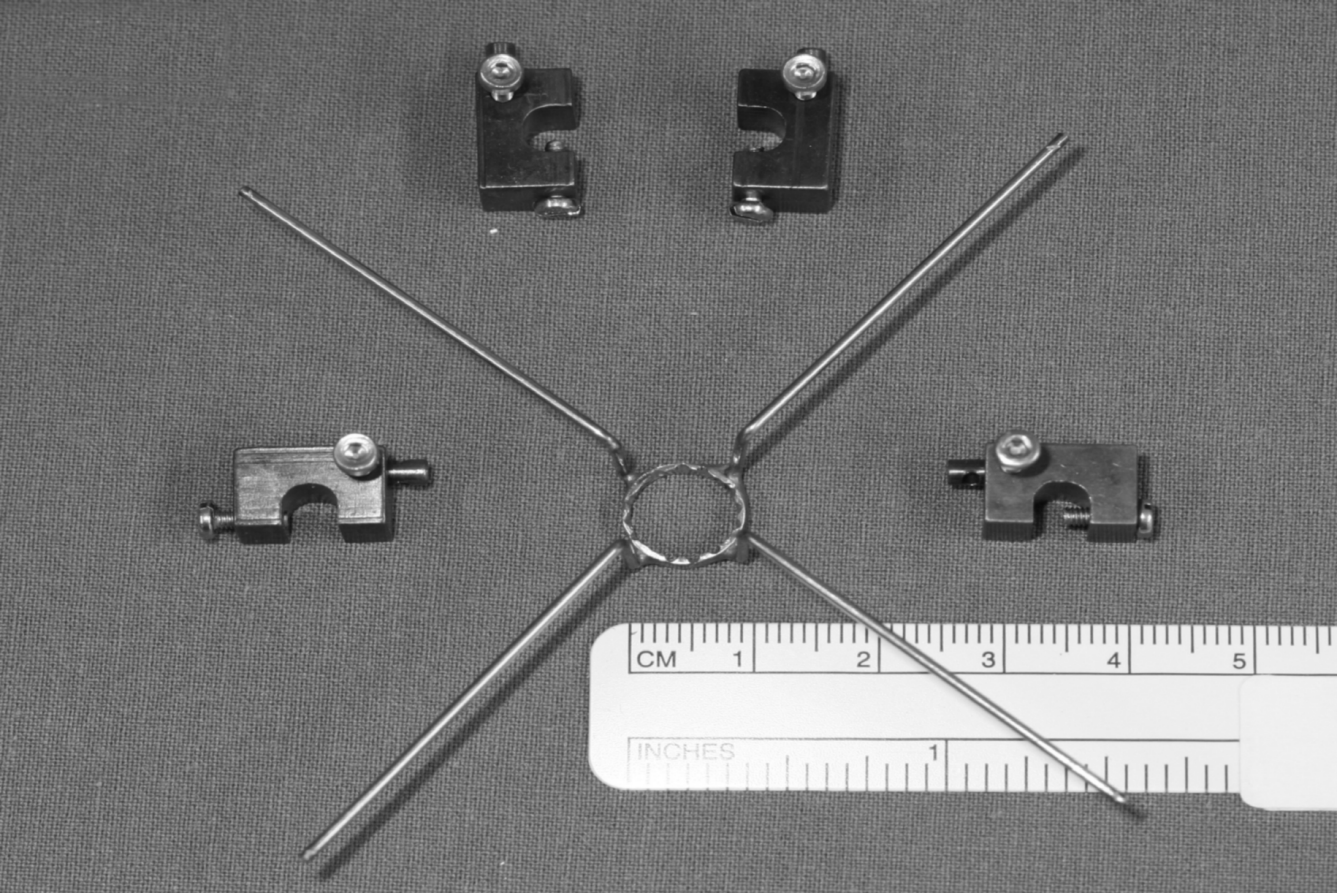
Stabilizer ring. Steel wire construction to support instruments during simulated ear surgery.

**Fig 3:**
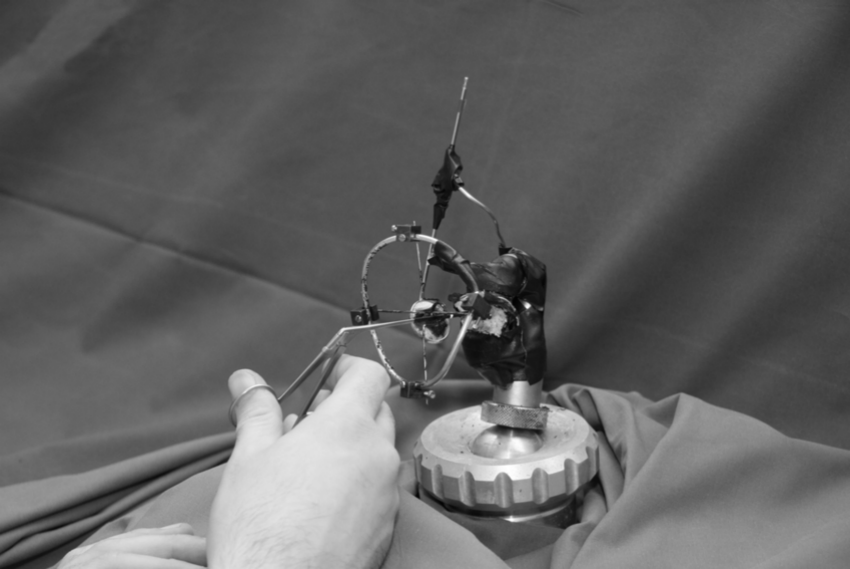
Measurement set-up. Measurement set-up with stabilizer ring attached to deissected temporal bone (microscope not shown). Note surgeon’s hand resting with fingers V and IV on an armrest during manipulation with the forceps.

An otosurgical microscope and otologic microinstruments were utilized: forceps and a pick(Karl Storz GmbH& Co. KG, Tuttlingen, Germany; catalog number: 221100 and225204) (Fig 1). The instruments were tested in different measurement protocols designed to simulate intraoperative conditions. Participants in the study performed basic surgical steps alternately using the pick and forceps. Overall, 10 different exercises were performed under visual control with the microscope (Table 1). Each session of measurements lasted for 10 s. There were several training trials to allow the subjects to become comfortable with the task but these were not included in the analysis. Some exercises traced freehand tremor while the test person was asked to hold an instrument above a fixed point on the promontory, without touching it (measurements No. 1, 2 and 7). Dynamic tasks consisted of either touching a point on the promontory with the pick (measurements No. 9 and 10), or grasping the long process of the incus with the forceps (No. 4, 5 and 6), 3–4 times each during the recording. These tasks approximated dissection exercises in middle ear surgery and the crimping process in stapes surgery, respectively. Tests were also performed with the instrument supported on a stabilizer tool (Figs 2–3) (No. 3, 6, 8 and 10). In the test battery, two-handed exercises were also included (No. 2 and 5). The surgeon’s hand (wrist) was always supported by an armrest during tasks (Fig 3). The effect of food abstinence, sleep deprivation or coffee consumption on tremor characteristics was not the subject of this study. However, physical exercise/sport up to 24 h before testing was an exclusion criterion. For the repeated measurements, we used an incomplete counterbalanced measures design using a “Latin Square” (Table 2). Counterbalancing was needed to ensure the validity of the experiment by eliminating factors changing the behavior of the test persons (for example, fatigue and stress). Every single task followed every other test once, allowing any carryover effects to be avoided during the statistical analysis.

**Table 1:** Tasks used in this study.

Task number	Task	Instrument
**1**	Pointing at a target (painted black point) on promontory, one-handed, without instrument support	Forceps
**2**	Pointing at a target (painted black point) on promontory, two-handed, without instrument support	Forceps
**3**	Pointing at a target (painted black point) on promontory, one-handed, with instrument support	Forceps
**4**	Grasping the long process of the incus 3–4 times, one-handed, without instrument support	Forceps
**5**	Grasping the long process of the incus 3–4 times, two-handed, without instrument support	Forceps
**6**	Grasping the long process of the incus 3–4 times, one-handed, with instrument support	Forceps
**7**	Pointing at a target (painted black point) on promontory, one-handed, without instrument support	Pick
**8**	Pointing at a target (painted black point) on promontory, one-handed, with instrument support	Pick
**9**	Touching a target (painted black point) on promontory, one-handed, without instrument support	Pick
**10**	Touching a target (painted black point) on promontory, one-handed, with instrument support	Pick

**Table 2:** Incomplete counterbalanced measures design using a “Latin square” to determine the chronologic order of tasks.

Test person	Task 1	Task 2	Task 3	Task 4	Task 5	Task 6	Task 7	Task 8	Task 9	Task 10
**1**	1	2	10	3	9	4	8	5	7	6
**2**	2	3	1	4	10	5	9	6	8	7
**3**	3	4	2	5	1	6	10	7	9	8
**4**	4	5	3	6	2	7	1	8	10	9
**5**	5	6	4	7	3	8	2	9	1	10
**6**	6	7	5	8	4	9	3	10	2	1
**7**	7	8	6	9	5	10	4	1	3	2
**8**	8	9	7	10	6	1	5	2	4	3
**9**	9	10	8	1	7	2	6	3	5	4
**10**	10	1	9	2	8	3	7	4	6	5
**11**	1	2	10	3	9	4	8	5	7	6
**12**	2	3	1	4	10	5	9	6	8	7
**13**	3	4	2	5	1	6	10	7	9	8
**14**	4	5	3	6	2	7	1	8	10	9

The experiments were performed by 14 otolaryngologists from the same ENT department with different levels of experience in otology (Table 3). Due to the limited number of test persons, only two groups were created: group 1 (*n* = 10) consisted of ENT surgeons having only limited otologic experience in terms of years and number of surgical procedures performed per year; however, they were familiar with the use of the microinstruments in this study. Test persons in group 2 (*n* = 4) were experienced otosurgeons due to their primary type of practice within the last 10 years and their performance of a minimum of 100 otologic procedures per year.

**Table 3:** Otologic experience of test persons.

Test person	Age (years)	Gender	Otologic experience
**1**	29	M	1
**2**	29	F	1
**3**	35	F	1
**4**	34	M	1
**5**	34	F	1
**6**	31	F	1
**7**	41	M	2
**8**	61	M	2
**9**	32	F	1
**10**	35	M	1
**11**	42	M	2
**12**	40	M	2
**13**	28	M	1
**14**	28	F	1

1, less experienced; 2, experienced; M, male; F, female.

### Stabilizer ring

**Fig 4:**
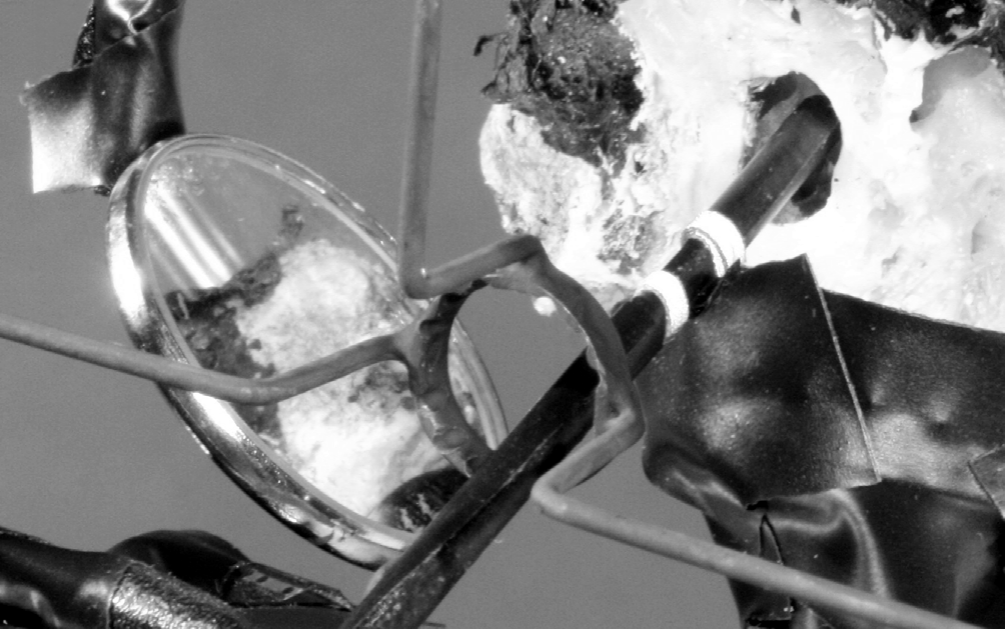
Instrument support. Forceps with markers for optical tracking. Simulation of intraoperative manipulation while the instrument is supported by the stabilizer ring. Note notches on the ring and the position of the mirror to allow detection of marker trajectories in three dimensions.

A stabilizer ring was used for IS (Figs 2–4). The tool consisted of a ring of 10 mm diameter with four orthogonal legs. The ring tool for resting otological microinstruments was mounted on bent alloy wires at a distance of 30 mm from the long process of the incus (Figs 3 and 4). The ring of the tool was positioned in the virtual axis of the outer ear canal to reproduce intraoperative conditions as far as possible. The microscopic view of the operating field was not obscured by the stabilizer. Microinstruments were placed on the inner circumference of the ring which had notches to prevent side-slip (drift) during manipulation (Fig 4).

### Video recording

Tremor measurements were carried out using a real-time passive marker-based analyzer of motion (PAM)[15]. Two passive markers (1.0 mm wide adhesive reflective strip) were attached to the distal portion of the instruments. The distance between the two markers was 3 mm, and the position of the distal marker was 15 mm away from the instrument tip (Fig 1). The handling of the microinstruments was not influenced by the markers as they had insignificant weight. The trajectories of the marker positions were determined from one direction with PAM. The system consisted of a digital video camera (DCR-HC23, Sony, Japan) with synchronized flashing infrared LEDs around the lens, IEEE1394 interface, and PC-based real-time recording software. The horizontal and vertical coordinates of the marker positions were determined at a sampling rate of 50 Hz. The frame of the camera (720×576 pixels) was vertically divided into two subframes by the computer. Each subframe, containing 360×576 pixels,showed the orthogonal projections of the distal portion of the instrument in a 30×30×48 mm volume of interest. One subframe contained direct reflections from the two markers attached to the instrument, the other contained indirect orthogonal reflections from the markers seen in a mirror placed at a 45° angle to the axis of the camera (Fig 4, S1 File). With this simple mirror system, it was possible to track the marker displacements in three dimensions. Removal of the lateral parts of the outer canal (see above) was necessary to visualize the displacement of the distal portion of the microinstruments. All of the metallic parts of the stabilizer and the otologic instruments in the measurement field were painted black or were covered with light absorbing adhesive tape to eliminate light reflections from the system. In this way, background noise during measurements could be diminished.

### Data analysis

Calibrated three-dimensional displacement–time series were analyzed with custom-made software, implemented in MATLAB 7.1 (Mathworks, Sherborn, Mass., USA).Data were filtered with a 5^th^ order Butterworth filter (bandpass 1.0–24 Hz).Frequency domain analysis was performed by computing the power spectral density with 0.05 Hz frequency bins. Finally, the 3-D position of the microinstrument’s tip was computed based on the axis of the instrument and the defined distance of the two markers from the tip. The displacement data with regard tothe methods of holding the instrument (conventional, TH, and IS), instrument type, and the surgical experience of the test persons were compared.

## Results

**Fig 5:**
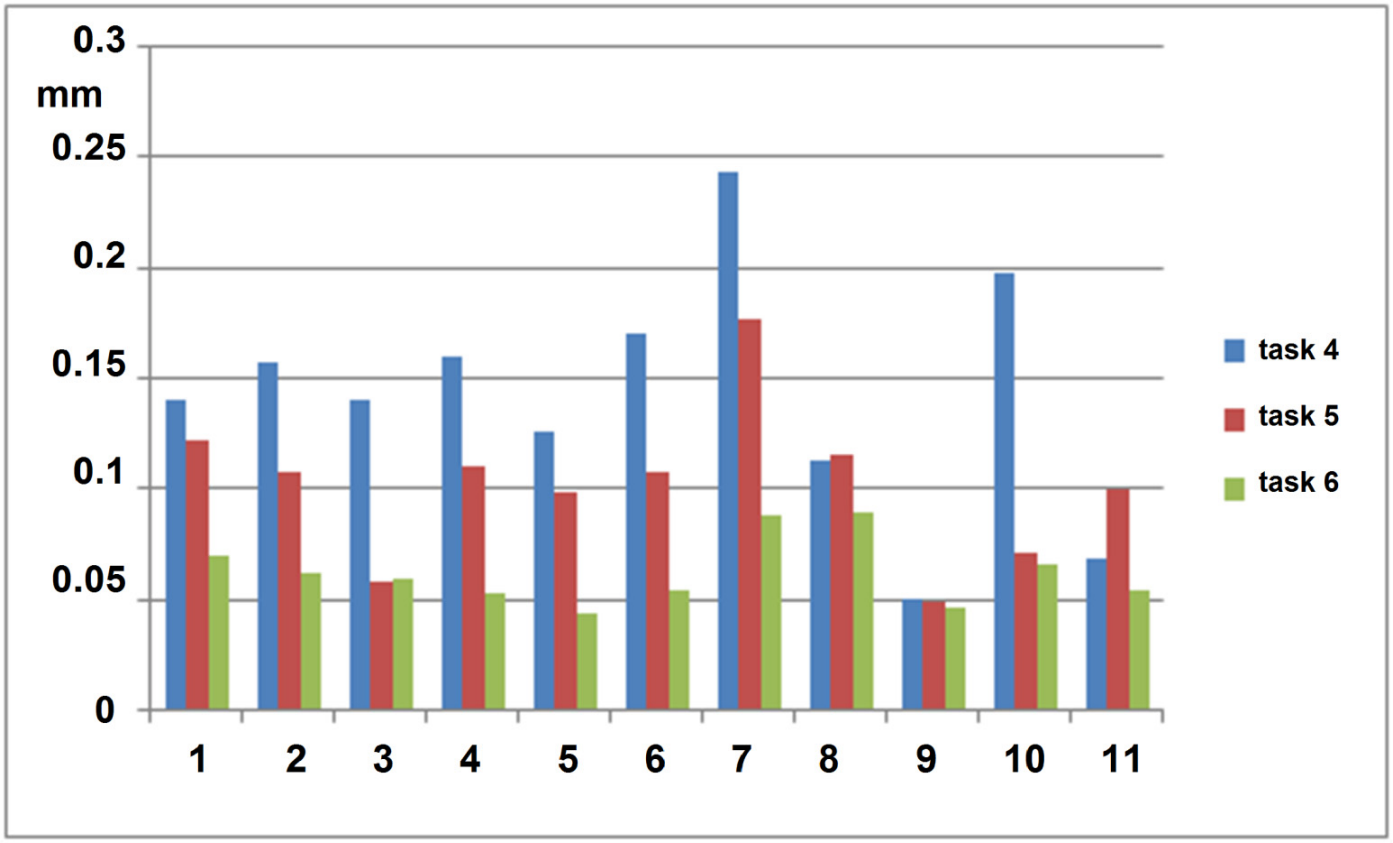
Marker trajectories. Root mean square (rms) displacement of 3-D marker trajectories of the forceps in tasks 4, 5 and 6.

Intrapersonal analysis of PAM data revealed that test persons having a greater tremor amplitude benefitted more from the IS technique as their tremor reduction was more pronounced than for test persons with less tremor (Fig 5). Irrespective of the extent of freehand tremor (measured at the instrument tip), the IS technique enabled a positioning accuracy in a range from 0.04 to 0.09 mm with the forceps (Fig 5) and from 0.03 to 0.11 mm with the pick (data not shown).

**Fig 6:**
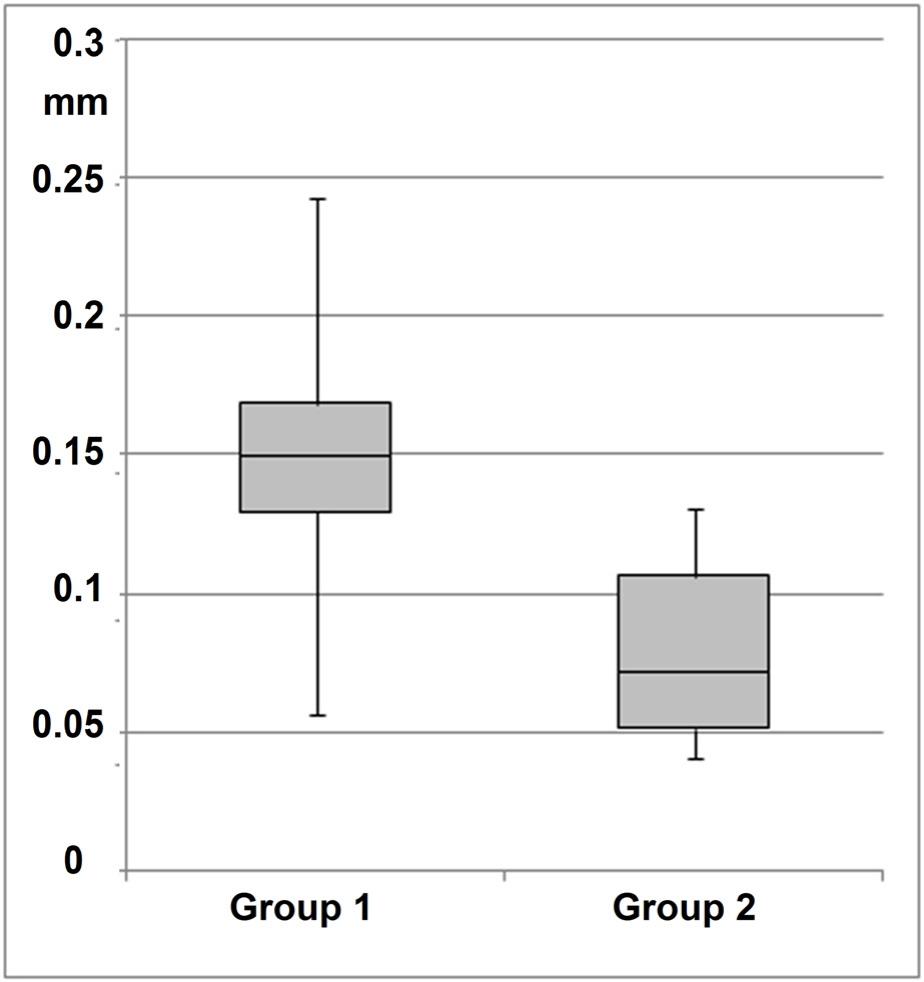
Manipulation with forceps. Experienced surgeons (group 2) have statistically significantly lower tremor amplitude (rms displacement of 3-D marker trajectories) than less experienced surgeons (group 1) (*t*: 0.008792) in task 4.

**Fig 7:**
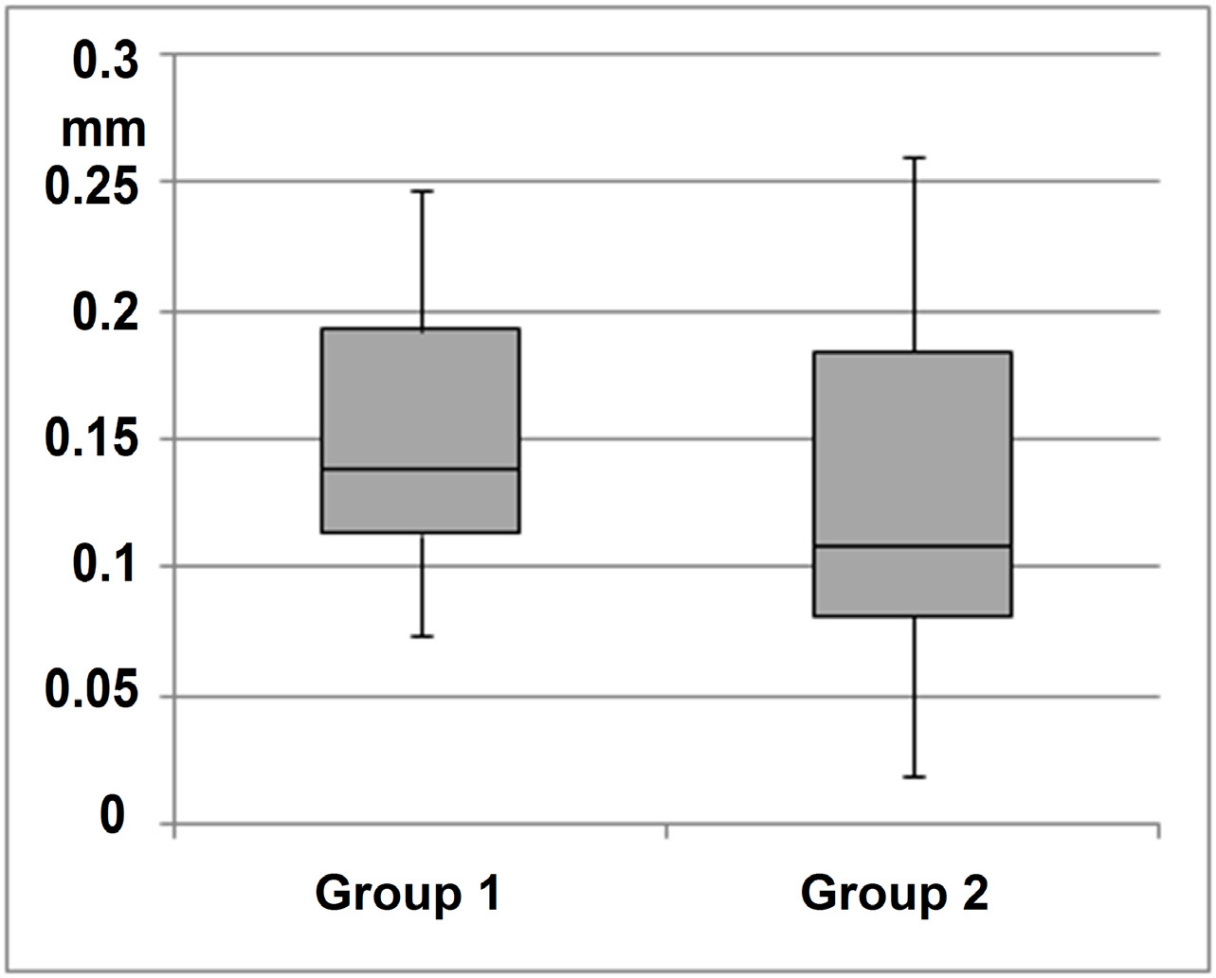
Manipulation with pick. Freehand manipulation with the pick (task 9) did not show a statistically significant difference (*t*: 0.476247) in tremor amplitude between less experienced and experienced surgeons.

Interpersonal analysis of freehand tremor indicated a significantly lower (*t*: 0.008792) tremor amplitude in group 2 (experienced surgeons) than in group 1 (less experienced surgeons) for task 4 (freehand grasping motions with forceps) (Fig 6). In contrast, freehand manipulation with the pick (task 9) showed no statistically significant difference (*t*: 0.476247) in tremor amplitude between less experienced and experienced surgeons (Fig 7).

**Fig 8:**
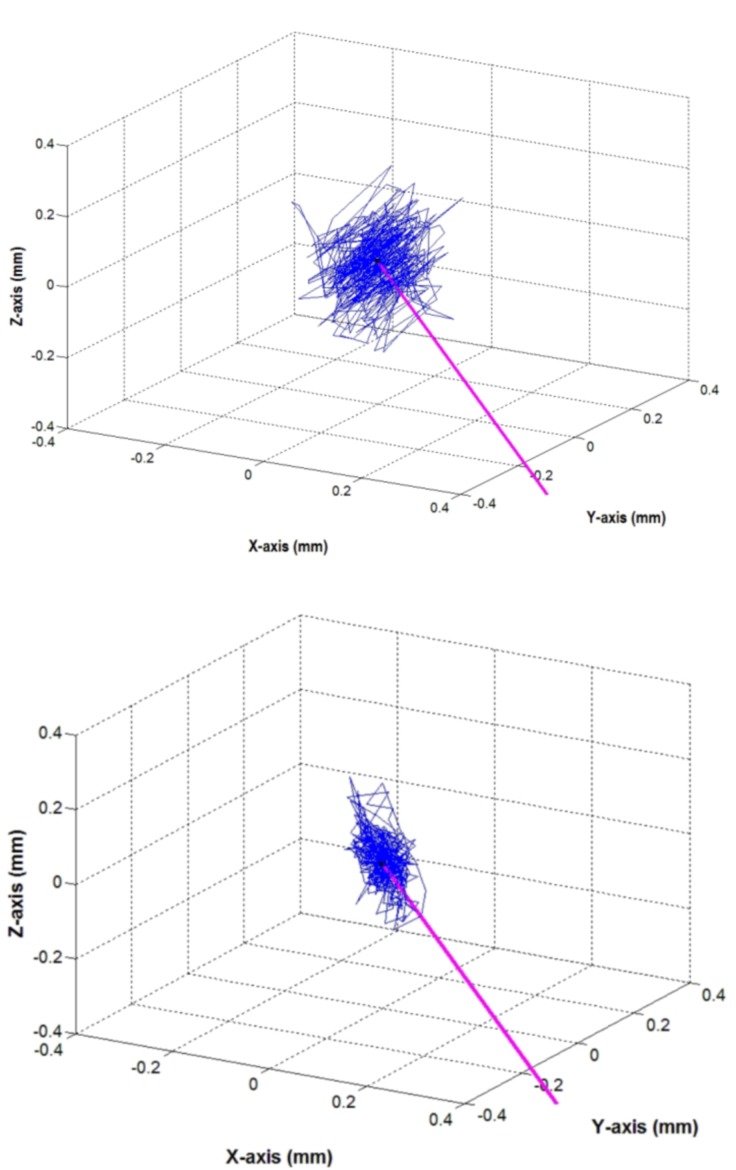
3-D marker trajectories. 3-D marker trajectories of the instrument tip (test person 4). Purple line: axis of the instrument (forceps). Top: one-handed uncompensated manipulation (task 4); bottom: manipulation with IS (task 6).

When using the forceps, comparison of marker trajectories showed a significant regression of trajectory field ranges with both two-handed and IS techniques relative to free one-handed manipulation in the dynamic tasks (tasks 4, 5 and 6) (Table 4). Root mean square (rms) displacement values with the IS technique decreased significantly more than with the two-handed manipulation technique (Table 4). Fig 8 demonstrates the extent of reduction in tremor amplitude measured at the instrument tip (one-handed uncompensated manipulation versus IS) for three axis.

**Table 4:** Paired sample statistics of one-handed, two-handed and IS holding technique (task 4, 5 and 6, respectively) with the forceps (rms of 3-D marker trajectories) (*n* = 11).

Paired differences								
					95% confidence			
Pairs	Pairs	Mean	Std. Deviation	Std. Error Mean	Lower	Upper	*t*-test	Significance (2-tailed)
**One-handed–Two-handed**	Task 4 –task 5	0.0409636	0.0446906	0.0134747	0.0109401	0.0709872	3.040	0.012
**One-handed—IS**	Task 4 –task 6	0.0800000	0.0493027	0.01468653	0.0468780	0.1131220	5.382	0.000
**Two-handed—IS**	Task 5 –task 6	0.0390364	0.0276888	0.0083485	0.0204348	0.0576379	4.676	0.001

Based on the test persons’ feedback, forceps-like otologic microinstruments are suitable for IS because they can be freely manipulated with grasping motions; however, the IS technique was reported to hinder free manipulation with needle-like instruments.

## Discussion

Surgical precision at the microscopic scale is adversely affected by tremor. Although hand trembling is not a significant factor for experienced surgeons, it can become a critical factor for otologists having less surgical expertise when performing surgical steps that require higher dexterity. For example, mechanical perforation of the stapes footplate or crimping of the stapes prosthesis are technically demanding procedures during which the physiological tremor-related risk is increased.

The spectrum of tremor magnitude varies widely among individuals. It depends greatly on the number of upper extremity joints which transmit movements from the last fixation point to the end of the microsurgical instrument. It is also influenced by the distance between the last supporting point and the surgical site. Tremor is also intensified by stress and fatigue [16,17].Typically, surgeons try to reduce tremor by fixing their forearms and hands, mainly with the ulnar side of the fifth digital ray, on a support table, armrest, speculum, or any part of the patient’s head [18].

However, previous measurements have shown that hand tremor causes a 0.2 to 0.5 mm trembling of surgical instruments (working length between 50 and 100 mm) even with conventional tremor reducing techniques [19]. Mürbe et al. validated the beneficial effect of two-handed manipulation on tremor amplitude [1].The present study investigated the impact of the TH and IS techniques on surgical precision. Both techniques proved to be superior to freehand manipulation. Ultimately, simple mechanical support of microinstruments was most effective for dampening unintentional movements of surgical instruments.

Intensive research is being undertaken on cancelling physiological tremor in different fields of microsurgery, enhancing the surgeon’s dexterity and the safety of operations. The use of micromanipulators is under investigation in order to bypass surgeon dependent impacts. This technology has already been investigated during cochlear implantation, tympanoplasty, and stapes surgery [2,3,6,7,11,13,14].Another trend is robot-assisted surgery and the development of surgical robots [5,8,12].The latter new technologies are superior in precision to skilled surgeons. However, tactile feedback is lost while operating with remotely controlled robotic instruments. Additionally, manipulating the robot requires a learning curve, just as with the traditional operative techniques [3]. Set-up time, operating time, and cost-effectiveness of robot technologies are still being monitored. Surgical skills are replaced by necessary skills for programing and planning of robotic surgery. The clinical impact on reduction in complications and improvement in surgical results, relative to conventional otologic microsurgery, are still the subject of debate. Additionally, these new technologies are not accessible for many institutions.

To the best of our knowledge, this is the first study to examine the effect of the TH and IS techniques on surgical precision with otologic instrumentation. The IS technique reduces motion caused by tremor and permits adequate instrument mobility during surgical manipulation (Fig 8) [20]. We adapted a simple and accurate measurement system and demonstrated that the TH and IS techniques effectively reduced unintentional instrument movements. The measurement data indicate that less experienced surgeons, particularly using the IS technique, can improve their positioning accuracy close to the level of a trained otologist. Accordingly, the IS technique is a simple but effective alternative to the complex systems with high investment costs mentioned above. The idea of instrument support is certainly not new [21–23]. Some surgeons push the forceps against the outer ear canal wall or operate through a speculum using it as a stabilizer. Indeed, optical tracking objectively demonstrates the significant benefit by attenuation of undesired instrument tip motion with the TH and IS techniques.

The laboratory set-up presented above provides feedback for otosurgeons about their tracing ability and tremor canceling performance. Hence, this system is a useful device for surgical skill assessment and a simulator for microsurgical training. Simulation of surgical steps in middle ear surgery has the benefit of training technical skills and decreases the risk to patients [24]. Performing crucial surgical steps in an otologic model may shorten the learning curve of trainees [25,26]. Since patient safety is a high priority, trainees should practice in a simulator during surgical training [24,26,27]. Furthermore, this simulator using 3-D tool tip tracking can be used for otologic microinstrument evaluation. The data collected may be useful to develop devices for robotic accuracy enhancement and positioning error compensation [28].

One of the commercially available temporal bone surgery simulators is the Voxel-Man ENT [29, 30, 31]. This system is based on virtual 3-D models derived from high-resolution CT data and is used for virtual temporal bone drilling. It has a force feedback device providing realistic haptic sensations and it is coupled to a virtual surgical navigation system for tracking instrument movements. Furthermore, the system allows trainees to upload their own work cases and provides automatic skill assessment with immediate feedback. However, besides drilling in temporal bone,otosurgery also integrates microsurgery of the ossicular chain and ear drum (tympanoplasty). In spite of the clear advantages of Voxel-Man ENT, the system is designed for simulating temporal bone dissection and not for simulating surgical manipulation with other instruments, i.e. forceps or pick used for tympanoplasty. The presented device with optical tracking enables the training of tympanoplasty techniques in the middle ear. As an alternative to cadaveric temporal bone specimens, a temporal bone model (for example, Phacon® Temporal Bone Patient [32]) can also be shaped and attached to this measuring system.

Nevertheless, the study has its methodological limitations as it only investigates the displacement of the surgeon’s otologic microinstrument and not the force of interaction between the instrument and target middle ear structures [22,33]. Measurement of positioning accuracy can only yield indirect data onthe forces and pressure exerted by the instrument tip. One can postulate that a decrease in tremor amplitude would reduce such forces and enable higher surgical precision. As a result, the risk of surgical damage, for example, incus luxation during prosthesis crimping or inadequate crimping, could be minimized. Consequently, further studies should aim to investigate the impact of the IS technique on transmitted forces during middle ear surgery.

Finally, the authors are aware of the fact that surgeons’ tremor is a factor influencing more the speed and precision of the surgery but not necessarily the outcome of otologic procedures. Nevertheless, the possible advantage of the IS technique is that it generally gives ear surgeons a “steady hand”, especially in difficult surgical situations.

## Conclusions

Simple tremor compensation techniques may offer trainees the potential to improve their positioning accuracy to the level of more experienced surgeons. Training in a temporal bone laboratory environment with optical tracking may help trainees acquire technical skills in middle ear surgery and potentially shorten the learning curve. Thus, simulated exercises of particular surgical steps should be integrated into surgical training.

## Supporting Information

S1 File Sample video of task 4. One of the test persons performs task 4. Video demonstrates marker trajectories in infrared light. Right side of the screen: real position of instrument markers. Left side of the screen: marker trajectories seen in mirror placed at a 45° angle to the axis of the camera.
(DV) Click here for additional data file.
